# Which type of fear of cancer progression contributes to the quality of life of Romanian cancer patients during the COVID-19 pandemic?

**DOI:** 10.3389/fpsyg.2023.1122339

**Published:** 2023-03-02

**Authors:** Éva Kállay, Flavia Medrea, Andrea Müller-Fábián, László Csaba Dégi

**Affiliations:** ^1^Faculty of Psychology and Educational Sciences, Babeș-Bolyai University, Cluj-Napoca, Romania; ^2^Faculty of Sociology and Social Work, Babeș-Bolyai University, Cluj-Napoca, Romania

**Keywords:** fear of cancer progression/recurrence, subjective QoL, COVID-19 pandemics, psycho-oncological care, Romania, LMIC

## Abstract

**Introduction:**

Fear of cancer progression (FoP) is one of the most frequently reported unmet needs invoked by the majority of cancer patients, which may significantly impair the quality of life (QoL) of patients. The major objective of the present cross-sectional study was to investigate the specificities of the relationship between different dimensions and intensity of FoP and different aspects of patients’ QoL during the COVID-19 pandemic in Romania.

**Methods:**

A nationwide sample of 330 participants completed a survey, including measures of demographic characteristics, medical variables, QoL, and FoP. Multivariate General Linear and Hierarchical Regression Models were conducted in order to assess the relationship between variables.

**Result:**

Our results indicate that less than a quarter of the sample experienced low, between 63 and 70% moderate, and 15% high levels of FoP. Our results also indicate that anxiety/worry related to the possibility of progression of the disease, and loss of independence produced significant differences with large effect sizes in all the dimensions of QoL.

**Discussion:**

Our results indicate that besides affective reactions, the fear of cancer survivors to lose independence, not being able to attend to their own lives, seems to be a considerable threat, especially in the context of Romanian health system which has difficulties in offering qualitative psychosocial care for cancer patients. The idea that patients will have to rely on others and may not function well independently, not being able to attend to their own lives, seems to be a considerable threat, next to the experienced affective reactions *per se*.

## Introduction

Diagnosis with cancer, the possible long- and short-term side effects of treatment produce significant changes in the lives of patients, as well as of their proximal and distal environments (families, friends, co-workers, etc.) (Singer, 2018), significantly affecting the quality of the patients’ physical, personal, professional, social, spiritual lives (Holland et al., 2010; Grassi and Riba, 2012). Approximately one-third of the cancer patients treated in acute-care experience intense symptoms of anxiety, depression, traumatic reactions, etc. (Mehnert et al., 2013; Nakash et al., 2014). Additionally to these psychological reactions, quite frequently, patients experience increases in existential worries/fears regarding the possible recurrence or progression of the disease, which further impacts the quality of their lives (QoL) (Fiszer et al., 2014; Curran et al., 2020). The relevant literature in psycho-oncology indicates that regardless the type of cancer and method of assessment, 24–97% of cancer survivors experience varying degrees of fear of cancer progression/recurrence (Mehnert et al., 2013; Simard et al., 2013), usually defined as “*fear, worry, or concern about cancer returning or progressing*” (Lebel et al., 2013) (p. 3).

According to the theoretical framework of the psycho-oncology literature fear of cancer recurrence/progression (FoP) is to some degree, a normal and adequate reaction to the threats and uncertainties survivors may experience at any stage of the illness- and survivorship-trajectory (Koch et al., 2013; Simonelli et al., 2017). Research indicates that FoP may persist years after the remission of the disease and cessation of treatment (Wagner et al., 2011). However, FoP may, in some cases attain maladaptive dimensions, leading to dysfunctional behavioral, cognitive, and emotional responses (Gotze et al., 2019). If these, otherwise realistic fears, become long-lasting and exaggerated, they may additionally contribute to the deterioration of the cancer survivors’ QoL (Herschbach and Dinkel, 2014). Since worldwide FoP is one of the most frequently cited unmet needs reported by the majority of cancer survivors (Crist and Grunfeld, 2013), research has intensified in this area to identify the most important risk and protective factors of this psychological phenomenon (for more, see Shay et al., 2016; Simonelli et al., 2017). Psycho-oncological research indicates that QoL is significantly associated with survivorship rates (Vodermaier et al., 2017; van Amelsfoort et al., 2022). Consequently, one of the most important factors in the ebb and flow of the FoP in the illness-trajectory is the certainty of access to treatment and the periodic follow-up examinations (Koral and Cirak, 2021).

## Background

The COVID-19 pandemic significantly changed the conditions and quality of human life worldwide, representing a significant source of distress, anxiety, uncertainty, loneliness, and confusion to the general population (Peteet, 2020). Obviously, this epidemic had a much deeper effect on cancer patients and survivors (Brivio et al., 2020). Besides the usual distress accompanying the entire oncological experience, cancer survivors had to additionally confront with the increased risk of contamination due to suppressed immune functioning (Gheorghe et al., 2021), and the restricted access to treatment and periodic follow-ups, which in many cases significantly increased the FoP (de Azambuja et al., 2020).

Economically, Romania is considered to belong among the developing countries LMIC (Low-Middle Income Countries) (WorldBank, 2022), with the second lowest share of GDP/capita dedicated to healthcare in the European Union (European Commission, 2021). Furthermore, Romania is characterized by a not very well-supported development of psychosocial oncology care where funding is frequently donor-dependent (Grassi et al., 2016). Consequently, it is not farfetched to expect that the situation of Romanian cancer patients and survivors during the COVID-19 pandemic has become even more dramatic, than in non-pandemic periods of life.

Empirical literature indicates that those cancer patients/survivors who experience higher levels of FoP indicate significantly lower levels of personal, professional, social, etc. QoL, impaired cognitive functioning (e.g., difficulties making plans for future due to the increased levels of uncertainty), hypochondria, excessive use of healthcare services, development of anxiety and depressive disorders, etc. which further on may affect their physical functioning as well (Hart et al., 2008; Lebel et al., 2013; Cincidda et al., 2022a,b). The increasingly high survival rates and life expectancy reclaim the thorough inquiry of the relationship between these two constructs (Shin et al., 2017).

### Purpose

Thus, the major objective of the present study was to conduct a more nuanced investigation regarding the relationship between different dimensions and intensities of FoP and different aspects of the QoL during the COVID-19 pandemic, using the STROBE guidelines for cross-sectional studies.

### Major objective

We expect that different degrees of FoP-dimensions will produce significant differences in the different facets of cancer patients’ QoL. This objective was strongly justified especially if we take into consideration the specific life-conditions created during the COVID-19 pandemic (frequent disruptions in the patients’ access to healthcare services, pending or suspended medical appointments, etc.), and that in Romania, the oncological care is frequently donor-dependent.

## Materials and methods

The CANPRIM project is the first nationwide study to address psychosocial burden, distress, and pathways among outpatient cancer patients in primary care in Romania. This questionnaire-based survey used an exploratory, descriptive, and cross-sectional approach.

Depending on tumor location, a heterogeneous, mixed sample of outpatient oncology patients was studied, comprising a nationwide sample of 330 individuals registered with public and private primary care providers in 34 of 41 counties, without restriction by cancer type (solid or hematologic) or stage. To be eligible for the study, outpatient participants had to be at least 18 years of age, provide informed consent, be mentally and physically capable of completing the questionnaire, and have functional digital literacy to perform the computerized measurement. Data collection was conducted *via* the CANPRIM application and by completing a paper-pencil questionnaire per participant’s choice in an anonymous and confidential process with coded responses. We had to use multiple strategic approaches (i.e., collaboration with primary care providers, collaboration with state and private medical oncology institutions, collaboration with psycho-oncologists and NGOs, and digital assessment tool) to collect data because our research was conducted in the context of the COVID-19 pandemic during a consecutive 8-months period in 2021. This study was performed in line with the principles of the Declaration of Helsinki. Approval was granted by Institutional Review Board (IRB) approval 16.260/30.10.2020 by the Scientific Council of Babeș-Bolyai University of Cluj-Napoca, Romania. Therefore, this study met all medical and research ethics requirements.

### Participants

Our study included a nationwide targeted sample of 330 Romanian cancer outpatients from 34 counties (out of 41 in total) assessed during May 2020 to September 2021. An *a priori* power analysis (G*Power 3.1; Faul et al., 2009) indicated that total sample size of 188 participants was required for a moderate effect size (*f*^2^ = 0.15), *α* = 0.05, 1-β = 0.85. After cleaning the database, we remained with the complete results of 313 participants (236 female-75.4%, and 77 male-24.6%), suffering of breast, uterine, colon and rectal, lung, prostate, bladder, oral, metastatic, and other oncological diseases. None of the patients received incentives for their participation in the study.

### Instruments

#### Demographic characteristics

Demographis characteristics included gender, age, level of education, marital status, and self-assessed socioeconomic status (SES).

#### Medical variables

Based on patients’ medical records and data sharing protocols endorsed by physicians, tumor location, tumor severity, the extent of disease, previous surgery, radiation, or chemotherapy, and time since oncology treatment began were recorded by trained researchers.

#### Quality of life

Quality of life was measured with the 30-item EORTC QLQ -C30 questionnaire (Aaronson et al., 1993). The EORTC QLQ-C30 offers information regarding the impact of the oncological disease and related treatments on the daily lives of cancer patients. Our study investigated the following parts of QLQ-C30: five functional subscales (physical, role, emotional, cognitive, and social functioning), financial difficulties (single-item). We decided to investigate the quality of the patients’ financial lives as well, since during the COVID-19 pandemic, due to the implemented health restrictions, the access to financial resources became scarcer than before (OECD, 2020; Sagan et al., 2021). The scales and single-item measures all have a range of values from 0 to 100. A high scale value indicates a higher response level. The psychometric properties of the EORTC are satisfactory, with Cronbach’s alpha ranging between 0.52 and 89 (Aaronson et al., 1993).

#### Fear of cancer progression

Fear of progression was assessed with the 43-item self-report Fear of Progression Questionnaire (FoP-Q) (Herschbach et al., 2005). We opted to use the FoP-Q because it offers the possibility of a multidimensional approach of the construct, comprising 5 subscales: *affective reactions* (anxiety/nervousness related to the progression/recurrence of the disease), *partnership/family* (fear/worry that illness will endanger the relationships within the family/partner, loss of sexual attractiveness, fear that the partner will leave due to the implications of the illness), *occupation* (anxiety/worry related to the inability to continue work due to illness, to financially support oneself, worry to cope with work-related demands), and *coping* (successful dealing with anxiety/fears/worries, seek help from professionals/family, optimism), *loss of independence* (the inability to: pursue hobbies and attend personal hygiene, the necessity to rely on others, not being accepted by others). The FoP-Q uses a 5-point Likert scale, higher scores indicating higher FoP levels. This questionnaire has excellent psychometric properties (Cronbach’s alpha over 0.70) (Herschbach et al., 2005).

## Results

In order to investigate our research questions, we conducted a correlation and hierarchical regression analysis. Table 1 presents descriptive statistics of the assessed variables, with the 3 categories of FoP (low, medium, and high), obtained after calculating the mean +/− one Standard deviation. Our results indicate that below 20% of the cancer patients/survivors indicate low, and over 11% (11.30–16.33%) high levels of FoP.

**Table 1:** Descriptive statistics (means, standard deviations, score ranges), with percentages of patients attaining low-medium-high levels of FoP.

		*N*	Min	Max	Mean	SD	Low	Medium	High
1	FoPQ_Affective_reactions	279	13	65	32.16	10.98	18.43%	66%	15.57%
2	FoPQ_Partnership_Family	284	7	34	15.27	5.30	18.37%	70.31%	11.30%
3	FoPQ_Occupation	287	7	35	14.78	6.88	16.54%	69.71%	13.75%
4	FoPQ_Coping	287	9	43	30.10	6.09	17%	68%	15%
5	FoPQ_Loss_of_Independence	303	7	35	15.29	6.14	20%	63.67%	16.33%
6	EORTC_QLQ_30_Physical_Functioning	311	0	100	66.10	21.44			
7	EORTC_QLQ_30_Role_Functioning	312	0	100	66.29	30.78			
8	EORTC_QLQ_30_Emotional_Functioning	309	0	100	67.40	26.82			
9	EORTC_QLQ_30_Cognitive_Functioning	311	0	100	73.15	26.60			
10	EORTC_QLQ_30_Social_Functioning	309	0	100	66.34	29.51			
11	EORTC_QLQ_30_Financial_Difficulties	308	0	100	42.20	33.82			

Next, in order to investigate possible differences in the assessed variables depending on low-medium-high levels of FoP, we performed Multivariate General Linear models (MGL), and applied *post hoc* Hochberg GT2 (significant differences are presented in Table 2).

**Table 2:** Multivariate general linear models for EORTC subscales, depending on low-medium-high levels of FoP subscales.

EORTC	FoPQ	*N*	Mean	Std. D	*F*	Sig	Partial η^2^
EORTC_Physical	FoPQ_Affective						
	Low	52	76.25*	19.25	24.41	**0.000**	0.150
	Medium	183	68.34*	19.44			
	High	44	48.78*	22.43			
	FoPQ_Partnership/Family						
	Low	52	70.35	22.92	8.26	**0.000**	0.056
	Medium	199	68.09	19.56			
	High	32	53.12	22.36			
	FPQ_Occupation						
	Low	48	73.02	19.11	3.87	**0.022**	0.027
	Medium	198	66.59	21.31			
	High	40	60.41	23.17			
	FoPQ_Loss of Independence						
	Low	61	76.96	19.49	15.76	**0.000**	0.095
	Medium	191	65.83	19.92			
	High	50	55.63	21.14			
EORTC_Role	FoPQ_Affective						
	Low	52	77.56	27.39	15.06	**0.000**	0.098
	Medium	183	69.30	28.87			
	High	44	46.59	30.41			
	FoPQ_Partnership/Family						
	Low	52	73.07	27.44	6.03	**0.003**	0.041
	Medium	199	68.34	30.38			
	High	32	50.52	32.65			
	FoPQ_Occupation						
	Low	48	78.81	26.34	5.86	**0.003**	0.040
	Medium	198	67.42	30.74			
	High	40	57.08	29.20			
	FoPQ_Loss of Independence						
	Low	61	80.05	25.24	15.39	**0.000**	0.093
	Medium	191	67.45	29.53			
	High	50	49.33	31.57			
EORTC emotional	FoPQ_Affective						
	Low	52	85.09	16.19	45.74	**0.000**	0.249
	Medium	183	69.50	22.97			
	High	44	40.71	29.10			
	FoPQ_Partnership/Family						
	Low	52	75.64	25.66	10.80	**0.000**	0.072
	Medium	199	69.66	23.44			
	High	32	50.00	34.71			
	FoPQ_Occupation						
	Low	48	79.34	19.14	8.95	**0.000**	0.059
	Medium	198	67.36	26.84			
	High	40	55.62	30.45			
	FoPQ_Coping						
	Low	49	70.57	27.22	4.35	**0.014**	0.030
	Medium	193	64.04	26.88			
	High	44	76.32	24.10			
	FoPQ_Loss of Independence						
	Low	61	84.01	16.96	33.19	**0.000**	0.182
	Medium	191	67.94	24.30			
	High	50	46.50	30.26			
EORTC cognitive	FoPQ_Affective						
	Low	52	82.05	23.99	16.55	**0.000**	0.107
	Medium	183	76.41	23.25			
	High	44	54.54	33.40			
	FoPQ_Partnership/Family						
	Low	52	78.20	25.89	4.21	**0.016**	0.029
	Medium	199	75.37	24.83			
	High	32	62.50	28.71			
	FoPQ_Occupation						
	Low	48	84.37	19.57	6.58	**0.002**	0.044
	Medium	198	73.73	26.29			
	High	40	64.58	29.03			
	FoPQ_Loss of Independence						
	Low	61	84.15	22.24	13.87	**0.000**	0.085
	Medium	191	74.69	24.71			
	High	50	59.33	28.20			
EORTC social	FoPQ_Affective						
	Low	52	74.35	24.57	15.03	**0.000**	0.099
	Medium	183	69.85	27.57			
	High	43	46.12	31.46			
	FoPQ_Partnership/Family						
	Low	52	69.87	25.35	9.77	**0.000**	0.066
	Medium	199	69.93	27.60			
	High	31	46.23	35.14			
	FoPQ_Occupation						
	Low	48	79.16	23.19	7.43	**0.001**	0.050
	Medium	197	67.68	28.80			
	High	39	55.55	32.29			
	FoPQ_Loss of Independence						
	Low	61	78.68	25.84	18.33	**0.000**	0.110
	Medium	191	67.97	27.72			
	High	48	46.52	31.12			
EORTC financial	FoPQ_Affective						
	Low	52	26.28	31.19	14.85	**0.000**	0.098
	Medium	180	38.51	32.83			
	High	44	61.36	28.70			
	FoPQ_Partnership/Family						
	Low	51	39.86	31.28	6.10	**0.003**	0.042
	Medium	197	38.40	33.95			
	High	32	60.41	31.03			
	FoPQ_Occupation						
	Low	48	20.83	28.86	15.02	**0.000**	0.097
	Medium	196	41.66	32.96			
	High	39	58.11	31.26			
	FoPQ_Loss of Independence						
	Low	61	24.59	28.48	20.47	**0.000**	0.122
	Medium	188	41.31	33.08			
	High	50	63.33	30.30			

*p < 0.05.

Our results indicate that small to medium size effects were produced in the following cases: low-medium-high levels of fear regarding family/partnership problems produced significant differences in the quality of physical [*F*(_2,283_) = 8.26, *p* = 0.000, partial η^2^ = 0.056], role [*F*(_2,283_) = 6.03, *p* = 0.003, partial η^2^ = 0.041], cognitive [*F*_(2,283)_ = 4.21, *p* = 0.016, partial η^2^ = 0.029], social [*F*(_2,283_) = 9.77, *p* = 0.000, partial η^2^ = 0.066], and financial [*F*(_2,283_) = 6.10, *p* = 0.003, partial η^2^ = 0.042] functioning; low-medium-high levels of occupation-related fears produced significant differences in the quality of physical [*F*(_2,283_) = 3.87, *p* = 0.022, partial η^2^ = 0.027], role [*F*(_2,283_) = 5.86, *p* = 0.003, partial η^2^ = 0.040], emotional [*F*(_2,283_) = 8.95, *p* = 0.000, partial η^2^ = 0.059], cognitive [*F*(_2,283_) = 6.58, *p* = 0.002, partial η^2^ = 0.044], and social [*F*(_2,283_) = 7.43, *p* = 0.001, partial η^2^ = 0.050] functioning, while low-medium-high levels of FoP_coping (successful dealing with anxiety/fears/worries) produced significant differences only in the quality of emotional functioning [*F*(_2,283_) = 4.35, *p* = 0.014, partial η^2^ = 0.030].

Medium to large effect sizes were produced by: low-medium-high levels of anxiety related to the disease (FoP_affective reactions) produced significant differences in the quality of physical [*F*(_2,283_) = 24.41, *p* = 0.000, partial η^2^ = 0.15], role *p*[*F*(_2,283_) = 15.06, *p* = 0.000, partial η^2^ = 0.098], emotional [*F*(_2,283_) = 45.74, *p* = 0.000, partial η^2^ = 0.249], cognitive [*F*(_2,283_) = 16.55, *p* = 0.000, partial η^2^ = 0.107], social [*F*(_2,283_) = 15.03, *p* = 0.000, partial η^2^ = 0.099], and financial [*F*(_2,283_) = 14.85, *p* = 0.000, partial η^2^ = 0.098] functioning; low-medium-high levels of fear regarding the way the illness may affect the relationship with the partner produced significant differences in the emotional [*F*(_2,283_) = 10.80, *p* = 0.000, partial η^2^ = 0.072], and social [*F*(_2,283_) = 9.77, *p* = 0.000, partial η^2^ = 0.066] dimensions of the QoL. Different levels in fear related to occupational changes produces by the illness produce significant differences in the emotional [*F*(_2,283_) = 8.95, *p* = 0.000, partial η^2^ = 0.059], and financial [*F*(_2,283_) = 15.02, *p* = 0.000, partial η^2^ = 0.097] dimensions of the quality pf life. Finally, low-medium-high levels of fear related to loss of independence produce significant differences in the following dimensions of the QoL: physical [*F*(_2,283_) = 15.76, *p* = 0.000, partial η^2^ = 0.095], role [*F*(_2,283_) = 15.39, *p* = 0.000, partial η^2^ = 0.093], emotional [*F*(_2,283_) = 33.19, *p* = 0.000, partial η^2^ = 0.182], cognitive [*F*(_2,283_) = 13.87, *p* = 0.000, partial η^2^ = 0.085], social [*F*(_2,283_) = 18.33, *p* = 0.000, partial η^2^ = 0.110], and financial [*F*(_2,283_) = 20.47, *p* = 0.000, partial η^2^ = 0.122].

Next, in order to identify the association patterns between variables, we conducted zero-order correlation analyses. Results are presented in Table 3. Our results indicate that the affective component of FOPQ is significantly correlated with all components of subjective well-being (physical_functioning: *r* = −0.43, *p* < 0.01; role_functioning: *r* = −0.32, *p* < 0.01; emotional_functioning: *r* = −0.56, *p* < 0.01; cognitive_functioning: *r* = −0.33, *p* < 0.01; social_functioning: *r* = −0.27, *p* < 0.01; financial difficulties: *r* = 0.31, *p* < 0.01). Partnership/Family is also significantly associated with physical_functioning: *r* = −0.16, *p* < 0.01; role_functioning: *r* = −0.20, *p* < 0.01; emotional_functioning: *r* = −0.30, *p* < 0.01; cognitive_functioning: *r* = −0.18, *p* < 0.01; social_functioning: *r* = −0.21, *p* < 0.01; and financial difficulties: *r* = 0.22, *p* < 0.01. Similar association patterns may be observed in the case of: occupation (physical_functioning: *r* = −0.18, *p* < 0.01; role_functioning: *r* = −0.20, *p* < 0.01; emotional_functioning: *r* = −0.28, *p* < 0.01; cognitive_functioning: *r* = −0.18, *p* < 0.01; social_functioning: *r* = −0.21, *p* < 0.01; financial difficulties: *r* = 0.27, *p* < 0.01), loss of independence (physical_functioning: *r* = −0.41, *p* < 0.01; role_functioning: *r* = −0.37, *p* < 0.01; emotional_functioning: *r* = −0.52, *p* < 0.01; cognitive_functioning: *r* = −0.31, *p* < 0.01; social_functioning: *r* = −0.34, *p* < 0.01; and financial difficulties: *r* = 0.77, *p* < 0.01); however, the coping component of FoP presents a weak, significant correlation only with financial difficulties (*r* = 0.13, *p* < 0.05).

**Table 3:** Zero-order correlations for all study measures.

		1	2	3	4	5	6	7	8	9	10	11	12
1	FOPQ_Total_Scores	1											
2	FOPQ_Affective_reactions	0.88**	1										
3	FOPQ_Partnership_Family	0.82**	0.63**	1									
4	FOPQ_Occupation	0.81**	0.61**	0.63**	1								
5	FOPQ_Coping	0.32**	0.05	0.21**	0.12*	1							
6	FOPQ_Loss_of_Independence	0.81**	0.72**	0.66**	0.63**	0.02	1						
7	EORTC_QLQ_30_Physical_Functioning	−0.34**	−0.43**	−0.16**	−0.18**	0.10	−0.41**	1					
8	EORTC_QLQ_30_Role_Functioning	−0.28**	−0.32**	−0.20**	−0.20**	0.01	−0.37**	0.60**	1				
9	EORTC_QLQ_30_Emotional_Functioning	−0.42**	−0.56**	−0.30**	−0.28**	0.08	−0.52**	0.42**	0.40**	1			
10	EORTC_QLQ_30_Cognitive_Functioning	−0.25**	−0.33**	−0.18**	−0.18**	−0.01	−0.31**	0.32**	0.28**	0.62**	1		
11	EORTC_QLQ_30_Social_Functioning	−0.27**	−0.30**	−0.21**	−0.21**	−0.01	−0.34**	0.31**	0.39**	0.47**	0.42**	1	
12	EORTC_QLQ_30_Financial_Difficulties	0.35**	0.31**	0.22**	0.27**	0.13*	0.37**	−0.28**	−0.33**	−0.43**	−0.37**	−0.46**	1

*p < 0.05, **p < 0.01. 1-FOPQ_Total_Scores, 2-FOPQ_Affective_reactions, 3-FOPQ_Partnership_Family, 4-FOPQ_Occupation, 5-FOPQ_Coping, 6-FOPQ_Loss_of_Independence, 7-EORTC_QLQ_30_Physical_Functioning, 8-EORTC_QLQ_30_Role_Functioning, 9-EORTC_QLQ_30_Emotional_Functioning, 10-EORTC_QLQ_30_Cognitive_Functioning, 11-EORTC_QLQ_30_Social_Functioning, 12-EORTC_QLQ_30_Financial_Difficulties.

Based on the association patterns presented in Table 2, we continued our investigation with conducting hierarchical multiple regression analyses (HMR) the components of Subjective QoL (EORTC_QLQ_30): Physical_Functioning, Role_Functioning, Emotional_Functioning, Cognitive_Functioning, Social_Functioning, and Financial_Functioning. For each criterion variable, in the first step of the HMR, we introduced demographic variables (age, gender, marital status, and stage of illness). Next, we entered stepwise the components of Fear_Of_Cancer_Progression (Affective_Reactions, Partnership_Family, Occupation, Coping, and Loss_of_Independence). After running the regression analyses, we selected those variables which significantly predicted the above-mentioned components of subjective QoL. Preliminary analyses were conducted to ensure no violation of the assumptions of normality, linearity and homoscedasticity. Results are presented in Table 4.

**Table 4:** Hierarchical regression model of the components of Subjective quality of life as criterion variables, with demographic variables (age, gender, marital status, stage of illness), and the components of fear of cancer progression as predictors.

	*R*	*R* ^2^	*R*^2^ change	*B*	SE	*ß*	*t*
EORTC_QLQ_C_30_Physical_Functioning							
Step 1
Age	0.17	0.03	0.03	−0.28	0.09	−0.17	−2.96(**)
Step 2	0.50	0.25	0.22				
Age FOPQ_Affective_Reactions				−0.41 −0.94	0.08 0.10	−0.25 −0.48	−4.83(***) −9.09(***)
EORTC_QLQ_C_30_Role_Functioning	FOPQ_Loss_of_Independence	0.37	0.14	0.14	−1.87	0.26	−0.37	−7.07(***)	EORTC_QLQ_C_30_Emotional_Functioning	Step 1
0.55							
0.30	0.30					FOPQ_Affective_Reactions				−1.31	0.12	−0.55	−10.74(***)	Step 2	0.56
0.32	0.01					FOPQ_Affective_Reactions	
		−1.33	0.12	−0.55	−11.01(***)	FOPQ_Coping	
		0.58	0.21	0.13	2.67(**)	Step 3	0.58
0.34	0.02					FOPQ_Affective_Reactions	
		−0.94	0.17	−0.39	−5.46(***)	FOPQ_Coping	
		0.54	0.21	0.12	2.53(*)	FOPQ_Loss_of_Independece	
		−0.97	0.31	−0.22	−3.09(**)	EORTC_QLQ_C_30_Cognitive_Functioning	FOPQ_Affective_Reactions	0.33	0.11	0.11	−0.81	0.13	−0.33	−5.97(***)	EORTC_QLQ_C_30_Social_Functioning	FOPQ_Loss_of_Independence	0.34	0.11	0.11	−1.64	0.26	−0.34	−6.23(***)	EORTC_QLQ_C_30_Financial difficulties	FOPQ_Loss_of_Independence	0.37	0.13	0.13
2.01	0.29	0.37	6.83(***)

*p < 0.05, **p < 0.01, ***p < 0.001.

Our results indicate that physical functioning was best explained by age [3.1%, *F*_(1,274)_ = 8.78, *p* = 0.003] and affective reactions of FOPQ [22.5%, *F*_(2,273_) = 47.05, *p* = 0.000], together predicting 25.6% of the variance in EORTC_QLQ_C_30_Physical_Functioning. Role_functioning was significantly predicted only by loss of independence [*F*_(1,300)_ = 50.07, *p* = 0.001], explaining 14.3% of the variance in Role_functioning. Investigating the predictors of EORTC_QLQ_C_30_Emotional_Functioning, we entered in the first model affective reactions [*F*_(1,265)_ = 115.54, *p* = 0.001], which explained 30.4% of the variance [*F*_(1,265)_ = 115.54, *p* = 0.001]. In step two, we introduced coping [*F*_(2,264)_ = 62.68, *p* = 0.001], which predicted 1.8% of the variance in Emotional functioning, and in step three, the loss of independence component of FOPQ, resulting in a significant model [*F*_(3,263)_ = 46.35, *p* = 0.001], explaining an additional 2.4% of the variance, the three variables together explaining a total of 34.6% variance in emotional functioning. Regarding cognitive functioning, only one model proved significant [*F*_(1,277)_ = 35.71, *p* = 0.000], with FOPQ_affective_reactions explaining 11.4% of variance. Furthermore, FOPQ_Loss_of_Independence proved the only significant predictor in both the social functioning [*F*_(1,298)_ = 38.92, *p* = 0.001, explaining 11.6% of the variance], and financial difficulties [*F*_(1,297)_ = 46.96, *p* = 0.001, explaining 13.7% of the variance] components of subjective well-being.

## Discussion

Diagnosis with cancer and the associated side effects of treatment produce significant changes both in the lives of patients, and their environments (Singer, 2018). Over one-third of the cancer patients experience intense symptoms of psychological malfunctioning, which significantly affects the quality of their physical, personal, professional, social, spiritual lives (Holland et al., 2010; Grassi and Riba, 2012). These debilitating psychological states are often aggravated by a more specific kind of fear, namely, existential worries regarding the possible recurrence/progression of the oncological disease. Even if FoP is a normal, natural phenomenon, which may persist after remission and cessation of treatment (Wagner et al., 2011; Simonelli et al., 2017), in a considerable number of cases, it may attain extreme forms which may further affect the patients’ QoL (Simard et al., 2013).

The major aim of the present study was to conduct a more nuanced investigation regarding the relationship between different dimensions and intensities of FoP and facets of the QoL during the COVID-19 pandemic. This objective is strongly justified especially if we take into consideration the specific life-conditions created during the COVID-19 pandemic (frequent disruptions in the patients’ access to healthcare services, pending or suspended medical appointments, reduced access to financial resources due to severe economic restrictions, etc.), in a LMIC country as Romania, where the oncological care is frequently donor-dependent.

Our results indicate that less than a quarter of the sample experienced low, between 63 and 70% moderate, and 15% high levels of FoP.

The results of our study are in line with previous investigations (e.g., Waters et al., 2013) indicating a strong relationship between different levels of fear (related to family/partnership problems, occupation, illness, and loss of independence) and quality of life (QoL) in different dimensions (physical, role, emotional, cognitive, social, and financial) (Dunn et al., 2015; Ban et al., 2021). Our study found that higher levels of fear in cancer patients in different areas significantly decreased the quality of life in the corresponding dimensions. For example, higher levels of fear related to the disease resulted in a significant decrease in physical, role, emotional, cognitive, social, and financial dimensions of the QoL. Similarly, higher levels of fear related to loss of independence resulted in a significant decrease in physical, role, emotional, cognitive, social, and financial dimensions of the QoL, results that were similar with those provided by previous research (Ortega et al., 2020; Xiang et al., 2020; Bandinelli et al., 2021). Moreover, the effect size for the relationship between fear and QoL was moderate to large. The most significant effect size was observed for anxiety related to the disease and fear of losing independence.

Furthermore, our results also suggest that the affective component of FOPQ strongly correlates with all components of subjective well-being, including physical, role, emotional, cognitive, and social functioning, as well as financial difficulties. A similar association pattern was also observed in partnership/family and occupation, while the loss of independence shows stronger correlations with all components of subjective well-being. The coping component of FoPQ presented a weak but significant correlation only with financial difficulties. These results highlight the importance of considering the affective component of FOPQ in assessing subjective well-being and its various components.

Finally, our results suggest that physical functioning is significantly influenced by age and affective reactions of FOPQ, explaining 25.6% of the variance in physical functioning. Role functioning is only significantly influenced by the loss of independence, explaining 14.3% of the variance. Emotional functioning is significantly influenced by affective reactions, coping, and loss of independence, together explaining 34.6% of the variance. Cognitive functioning is only significantly influenced by affective reactions of FOPQ, explaining 11.4% of the variance. Social functioning and financial difficulties are only significantly influenced by the loss of independence, explaining 11.6 and 13.7% of the variance, respectively.

In conclusion, our study found a strong relationship between different levels of fear in cancer patients and quality of life in various dimensions, with higher levels of fear leading to a decrease in the corresponding quality of life, similar to the results presented in, e.g., Ban et al. (2021) study conducted during the COVID-19 pandemic, and Gotze et al. (2019) pre-pandemic investigation. The affective component of FOPQ was found to be strongly correlated with all components of subjective well-being, except for financial difficulties, which showed a weak correlation. The results also showed that physical, role, emotional, cognitive, social, and financial dimensions of the quality of life were influenced by different factors, with the most substantial influences being affective reactions, loss of independence, and coping mechanisms. Overall, the findings highlight the importance of considering fear in cancer care and its affective component in assessing subjective well-being and its various components.

### Study limitations

Obviously, our study has several limitations that have to be taken into consideration when interpreting the obtained results. First of all, our sample was quite heterogeneous (different types of cancer); thus, due to the relatively low number of participants, we could not conduct a more nuanced analysis of the data. For the same reason, we could not take into consideration the period of time elapsed between diagnosis and assessment. Also, our research relies solely on the participants’ self-report which may to some degree be biased. Future research may focus on obtaining information from multiple resources (e.g., physicians, family members). Furthermore, because of methodological considerations, we could not establish real causal relationships, only association patterns. For future studies we propose a longitudinal, dynamic approach of the phenomenon, which would additionally take into consideration both type of cancer and time elapsed since diagnosis, thus obtaining a more accurate picture of the investigated phenomenon.

### Recommendations for practice and research

Our results evidence the combined importance of two major components of FoP: anxiety/nervousness related to the progression/recurrence of the disease, and the loss of independence due to the implications the illness and its short- and long-term side effects. The idea that patients will have to rely on others and may not function well independently, not being able to attend to their own lives, seems to be a considerable threat, next to the experienced affective reactions *per se*.

Given the potential impact of unmet needs and fear of numerous personal losses in cancer, research-based, early, and multi-pronged psychosocial and educational interventions tailored to high-risk situations and socioeconomic backgrounds should be offered to cancer patients at all stages of the disease, during treatment, and throughout survival (Holland, Bultz, and Network, 2007; Holland et al., 2010) to improve psychosocial well-being and QoL, associated with survival in some cancer patient groups (Vodermaier et al., 2017; van Amelsfoort et al., 2022). The proper information of the medical staff and healthcare professionals regarding the real medical and psychosocial needs of the cancer patients/survivors, and the implementation of intervention suggestions based on these results may both lessen dysfunctional symptoms and increase the chances of cancer survivorship.

## Conclusion

In summary, the major conclusion of our study suggests that in the case of the assessed sample of Romanian cancer patienst during the COVID-19 pandemic, the quality of *physical functioning* is best predicted by age and affective reactions (25.5%), *role functioning* by loss of independence (14.3%), *emotional functioning* by affective reactions, coping, and loss of independence (34.6%), *cognitive functioning* by affective reactions (11.4%), *social functioning* by loss of independence (11.6%), and *financial difficulties* by loss of independence (13.7%). Our results may have high relevance not only in the specific socioeconomic context of Romania, but also in the COVID-19 conditions, especially if we take into consideration the uncertainty regarding the evolution of the pandemic and the imminence of crisis situations worldwide. Thus, we consider that these results may benefit prevention and intervention programs that target the enhancement the quality of life of cancer patinets.

## Data availability statement

The raw data supporting the conclusions of this article will be made available by the authors, without undue reservation.

## Ethics statement

The studies involving human participants were reviewed and approved by Institutional Review Board (IRB) approval 16.260/30.10.2020 by the Scientific Council of Babeș-Bolyai University of Cluj-Napoca, Romania. The patients/participants provided their written informed consent to participate in this study.

## Author contributions

All authors listed have made a substantial, direct, and intellectual contribution to the work, and approved it for publication. All authors participated in all phases of this work. ÉK and LD made a major contribution to this manuscript, FM made a medium contribution, and AM-F made a minor contribution. All authors reviewed the manuscript.

## Funding

The research leading to these results received funding from the Ministry of Research, Innovation and Digitization grant, CNCS/CCCDI – UEFISCDI, project number PN-III-P1-1.1-TE-2019-0097, within PNCDI III.

## Conflict of interest

The authors declare that the research was conducted in the absence of any commercial or financial relationships that could be construed as a potential conflict of interest.

## Publisher’s note

All claims expressed in this article are solely those of the authors and do not necessarily represent those of their affiliated organizations, or those of the publisher, the editors and the reviewers. Any product that may be evaluated in this article, or claim that may be made by its manufacturer, is not guaranteed or endorsed by the publisher.

## Abbreviations

FoP: fear of cancer progression
QoL: quality of life
LMIC: low-middle income countries
SES: socioeconomic status
FoP-Q: fear of progression questionnaire
MGL: multivariate general linear models
HMR: hierarchical multiple regression analyses
